# Retraction Note: Different azithromycin protocols for management of preterm prelabour rupture of membranes: a randomized clinical trial

**DOI:** 10.1186/s12884-025-08076-z

**Published:** 2025-08-30

**Authors:** Laila Ezzat Abdelfattah, Rehab Abdelhamid Aboshama, Amr S. Abdelbadie, Mohamed H. Abulhasan, Mohamed A. Anan, Ibraheem I Abdelaal

**Affiliations:** 1 Associate Professor of Obstetrics and Gynecology, Department of Obstetrics and Gynecology, Faculty of Medicine, Fayoum University, Fayoum, Egypt; 2 Lecturer of Obstetrics and Gynecology, Department of Obstetrics and Gynecology, Faculty of Medicine, Fayoum University, Fayoum, Egypt; 3 Lecturer of Obstetrics and Gynecology, Department of Obstetrics and Gynecology, Faculty of Medicine, Aswan University, Aswan, Egypt; 4 Resident of Obstetrics and Gynecology, Department of Obstetrics and Gynecology, Faculty of Medicine, Aswan University, Aswan, Egypt; 5 Lecturer of Obstetrics and Gynecology, Department of Obstetrics and Gynecology, Faculty of Medicine, Assiut University, Assiut, Egypt

**Retraction Note to: BMC Pregnancy and Childbirth (2022) 22:869** 10.1186/s12884-022-05189-7

The Editor has retracted this article. After publication, concerns were raised over the discrepancy between data in the article and other existing studies. Further investigation uncovered inconsistencies between the information in the clinical trial registry and the article, such as the university conducting the study and gestational age of the foetuses. The authors failed to provide evidence that ethical approval had been received prior to the commencement of the study. Ibraheem I Abdelaal, Amr S. Abdelbadie, Laila Ezzat Abdelfattah and Mohamed H. Abulhasan disagree to this retraction. Rehab Abdelhamid Aboshama and Mohamed A. Anan did not respond to correspondence regarding this retraction.

